# Pleiotrophin/Midkine Pathway Is Dysregulated in a TDP‐43^A315T^ Mouse Model of Amyotrophic Lateral Sclerosis (ALS)

**DOI:** 10.1111/neup.70044

**Published:** 2026-01-28

**Authors:** Paloma Martínez‐Alesón, Cristina Benito‐Casado, Carmen María Fernández‐Martos, María José Polanco Mora

**Affiliations:** ^1^ Departamento de Ciencias Farmacéuticas y de la Salud, Facultad de Farmacia Universidad San Pablo‐CEU, CEU Universities, Urbanización Montepríncipe Madrid Spain; ^2^ Instituto Universitario de Estudios de Las Adicciones, Universidad San Pablo‐CEU, CEU Universities, Urbanización Montepríncipe Madrid Spain; ^3^ Wicking Dementia Research and Education Centre, College of Health and Medicine University of Tasmania Hobart Tasmania Australia; ^4^ Red de Investigación en Atención Primaria de Adicciones, Instituto de Salud Carlos III, MICINN and FEDER Madrid Spain

**Keywords:** amyotrophic lateral sclerosis (ALS), inflammation, midkine (MK), motor neuron disease (MND), pleiotrophin (PTN), spinal cord (SC)

## Abstract

Amyotrophic lateral sclerosis (ALS) is a fatal motor neuron disease (MND) characterized by progressive degeneration of both upper and lower motor neurons, along with skeletal muscles innervated by them. The identification of key molecules involved in disease pathology remains crucial for ALS, as no curative treatment is currently available. Pleiotrophin (PTN) and midkine (MK) are closely related, heparin‐binding cytokines with overlapping effects. These molecules have been shown to be neuroprotective by modulating neuroinflammation, supporting neuronal survival, growth, and differentiation, and enhancing synaptic strength and plasticity. Despite their reported neuroprotective properties, the involvement of PTN and MK signaling in ALS has not been previously investigated. In this study, we characterized the expression of the PTN/MK pathway in the lumbar spinal cords (SCs) of TDP‐43^A315T^ mice across different disease stages. We report a significant upregulation of *Ptn*, *Mdk*, and its receptor protein tyrosine phosphatase zeta (*Ptprz1*) mRNA levels at end‐stage of disease in the lumbar SC of TDP‐43^A315T^ mice compared with age‐matched wild‐type littermates. Protein levels of PTN and MK were also upregulated at end‐stage of disease. By immunofluorescence analysis, we also observed an upregulation of the immunostaining of both cytokines in neurons, astrocytes, microglia, and pericytes‐like structures at end‐stage of disease in the SC of TDP‐43^A315T^ mice. These findings open a new avenue to further study the potential role of the PTN/MK signaling axis in the pathogenesis of ALS.

**Trial Registration:** Animal Ethics Committee of the Hospital Nacional de Parapléjicos in Toledo (Spain): Approval No. 26/OH 2018

## 1 Introduction

Amyotrophic lateral sclerosis (ALS) is a neurodegenerative disease affecting 1.9 per 100 000 population worldwide. The disease typically manifests in individuals between 40 and 65 years of age, although earlier onset can also occur. As the disease progresses, the motor neuron (MN) loss leads to denervation of skeletal muscles, resulting in weakness, atrophy, and ultimately, muscle paralysis. As a result, most ALS patients die from respiratory failure, often within 5 years of diagnosis, due to impaired ventilation and aspiration pneumonia [1]. Currently, seven treatments for ALS have been approved by the US Food and Drug Administration (FDA), aiming to alleviate symptoms and slow the disease's progression, but with modest effects in the disease pathogenesis [2].

Around 10% of ALS cases are inherited, due to mutations in various unrelated genes, while the other 90% do not have a familial history. Although mutations in *TARDBP*, encoding transactivation response DNA‐binding protein of 43 kDa (TDP‐43), account for ~4% of familial and ~1% of sporadic ALS cases, TDP‐43 proteinopathy is implicated in nearly all forms of ALS and frontotemporal dementia. The pathological hallmark involves cytoplasmic accumulation of TDP‐43 aggregates in affected tissues, accompanied by hyperphosphorylation, ubiquitination, and mislocalization of the protein. These alterations disrupt key cellular processes, including RNA metabolism, proteostasis, and stress responses, thereby leading to neurodegeneration [3, 4, 5]. Additionally, MN damage is associated with neuroinflammatory responses in patients and in animal models of ALS, with proliferation and activation of astrocytes, microglia, and other peripheral immune cells, which can exacerbate MN degeneration [3, 6, 7, 8, 9].

Transgenic models expressing mutant forms of TDP‐43 have been generated to recapitulate hallmark features of both ALS and frontotemporal lobar degeneration (FTD) associated with TDP‐43 proteinopathy. One widely utilized line carries the human *TARDBP* gene harboring the A315T substitution (TDP‐43^A315T^) under the murine prion promoter and develops motor dysfunction and premature mortality [10, 11]. In this mouse model, exogenous TDP‐43^A315T^ protein shows the highest levels of expression in brain, spinal cord (SC), and testis, with lower levels in skeletal muscle. Specifically in the SC, TDP‐43^A315T^ is present in MNs, oligodendroglia, and astrocytes [10]. Cytoplasmic mislocalisation of TDP‐43, occasional ubiquitinated inclusions, disruption of nuclear TDP‐43 homeostasis, and downstream RNA‐metabolic changes have been reported [12, 13]. Concomitantly, degeneration of spinal and brainstem MNs, axonal loss, muscle denervation, and gliosis have been documented [14]. Importantly, astrocytic and microglial activation accompanies the neuronal injury, indicating a prominent neuroinflammatory component. Collectively, the TDP‐43^A315T^ transgenic mouse recapitulates key molecular and cellular pathologies of ALS/FTD‐TDP‐43 and thus constitutes a valuable experimental platform for studying TDP‐43 aggregation, neuronal vulnerability, and therapeutic targeting in MN degeneration [15].

Pleiotrophin (PTN) and midkine (MK) are secreted cytokines with ~50% of sequence homology that belong to the heparin‐binding growth factor family [16]. Both act as neurotrophic factors, often showing overlapping expression and function. PTN and MK promote neuronal survival, differentiation, and axonal growth during development and after injury [16]. MK protein expression levels are upregulated in biopsied muscle tissue during early stages of regeneration in patients with myopathies [17], and genetic ablation of this gene in mice delays peripheral nerves and muscle regeneration following sciatic nerve injury [18, 19]. Similarly, in animal models of SC injury (SCI) PTN is overexpressed at both mRNA and protein levels in areas close to the injury and has been shown to stimulate motor axon outgrowth in explant cultures [20, 21]. Both PTN and MK contribute to functional recovery after SCI, partly by mitigating inflammation that facilitates neuronal damage and by antagonizing the inhibitory effects of chondroitin sulfate proteoglycans in the lesion microenvironment [22, 23, 24]. In neurodegenerative diseases such as Parkinson's and Alzheimer's disease, PTN has been shown to modulate neuroinflammation and promote a neuroprotective microglial phenotype in response to injury in cell cultures and mice [25, 26, 27]. In ALS, MK has been identified in the pool of several neuroprotective factors secreted by human embryonic stem cell‐derived astrocytes following intrathecal transplantation into ALS‐SOD1^G93A^ mice, though its specific role was not studied in isolation [28].

The neurotrophic effects of PTN and MK are mediated through interactions with several cell surface receptors, including receptor protein tyrosine phosphatase (RPTP) β/ζ signaling pathways [29]. RPTPβ/ζ is highly expressed in the central nervous system (CNS), and binding of PTN to RPTPβ/ζ promotes myelination during development and remyelination after injury [30, 31, 32, 33], while MK binding to RPTPβ/ζ has been shown to be essential for MK‐induced neuron survival [34]. Despite the growing evidence supporting neuroprotective and anti‐inflammatory properties of PTN and MK, their potential role in ALS pathophysiology remains unexplored. In this study, we investigated the expression patterns of the PTN/MK pathway in the SC of TDP‐43^A315T^ transgenic mice, a well‐established model of ALS, across different disease stages. Our aim was to determine whether PTN and MK may play a contributory role in ALS pathogenesis since therapeutic approaches aimed at modulating neuroinflammatory processes are increasingly recognized as promising avenues. Advancing the identification of novel molecular targets involved in these mechanisms is pivotal to the development of effective interventions for ALS.

## 2 Materials and Methods

### 2.1 Animals

Animal care and experimental procedures were conducted in accordance with the Spanish Guidelines for the Care and Use of Animals for Scientific Purposes and were approved by the Animal Ethics Committee of the Hospital Nacional de Parapléjicos in Toledo (Spain) (Approval No 26/OH 2018). TDP‐43^A315T^ mice [10] and age and sex‐matched wildtype (WT) littermate controls were used in this study. Only males were included in this study. Expression of human TDP‐43 (*hTDP‐43 transgene*) was confirmed via polymerase chain reaction according to the manufacturer's protocol [10]. Mice were housed in filtered cages in a temperature‐controlled room with a 12‐h light/dark cycle with ad libitum access to water and food. To monitor disease progression and onset determination, all mice were weighed and assessed three times per week until the disease onset stage, after which they were checked daily in the morning until the disease end stage.

### 2.2 Perfusion and Tissue Collection

Animals were terminally anesthetized with sodium pentobarbitone (140 mg/kg, intraperitoneally) and transcardially perfused with either 0.01 M phosphate‐buffered saline (PBS) (pH 7.4) or 4% paraformaldehyde (PFA). The lumbar SC from each animal was immediately dissected, postfixed overnight in the same fixative solution, and then cryoprotected sequentially in 18% and 30% sucrose solutions overnight. Serial coronal cryosections (30 μm thickness) were cut on a cryostat (Leica CM 1850). For molecular biology experiments, separate lumbar SC samples were processed for either quantitative real‐time polymerase chain reaction (RT‐qPCR), immunoblotting or immunofluorescence analysis. Samples were stored at −80°C until further use.

### 2.3 RT‐qPCR

Total RNA was isolated from lumbar SC using the RNeasy Mini Kit (Qiagen), according to the manufacturer's instructions. Complementary DNA (cDNA) was synthesized from 500 ng of total RNA using the High‐Capacity cDNA Reverse Transcription Kit (Applied Biosystem). Gene expression levels were quantified by RT‐qPCR using the SYBR Green method on a 7900 HT Fast Real‐Time PCR System (Applied Biosystems) and the C100 Touch Thermal Cycler, CFX96 Real‐Time System (Biorad). The following primers (Invitrogen) were used: 18S‐F, 5′‐AGTCCCTGCCCTTTGTACACA‐3′; 18S‐R, 5′‐GCCTCACTAAACCATCCAATCG‐3′; PTN‐F, 5′‐AGCAGTTTGGAGCTGAGTG‐3′; PTN‐R, 5′‐CTGCCAGTTCTGGTCTTCAA‐3′; MK‐F, 5′‐TGGACCCGACTGCAAATAC‐3′; MK‐R, 5′‐ATTGTACCGCGCCTTCTTC‐3′; RPTPβ/ζ‐F, 5′‐GAACGGATTTGGCCGTATTG‐3′; RPTPβ/ζ‐R, 5′‐GTGAGTGGAGTCATACTGGAA‐3′. 18S rRNA was used as the endogenous control. Relative gene expression was calculated using the ∆∆*C*_t_ method.

### 2.4 Western Blotting (WB)

For protein expression analysis, SC from asymptomatic, onset and end‐stage mice (42–45 days, 65–70 days, and 95–100 days, respectively) were pulverized using pestle and mortar. Proteins were extracted from pulverized tissues using RIPA buffer containing a cocktail of phosphatase and protease inhibitors (ref 11836153001 and ref 4906837001, Roche, Basel, Switzerland). Protein concentration was determined by bicinchoninic acid assay (ref 23225, Thermo Fisher Scientific, Waltham, MA, United States). Equal amounts of protein were subjected to 12% tris‐HCl SDS‐PAGE. Gels were blotted onto PVDF membranes (GE Healthcare Life Sciences). Antibodies used were PTN (an anti‐PTN, mouse monoclonal, H‐6 clone, Cat. sc‐74 443, Santa Cruz Biotechnology, Dallas, Texas, USA, 1:200), MK (an anti‐MK, mouse monoclonal, A‐9 clone, Cat. sc‐46 701, Santa Cruz Biotechnology, Dallas, Texas, USA, 1:200), and β‐actin (an anti‐β‐actin, rabbit monoclonal, Cat. 4967, Cell Signaling, Danvers, Massachusetts, USA, 1:10 000). WB signals were detected using the enhanced chemiluminescence reagent (ECL Prime Western Blotting Detection Reagent, Cat. GERPN2235 GE Healthcare Life Sciences). Quantification was performed using ImageJ 1.45 software.

### 2.5 Immunofluorescence

Lumbar SC cryosections were attemperated for 1 h at room temperature (RT) and then incubated with blocking solution containing 10% normal goat serum, 5% bovine serum albumin, and 5% milk in PBS. The incubation with the primary antibodies for detection of NeuN (an anti‐NeuN, chicken polyclonal, Cat. 266006, Synaptic System, Goettingen, Germany, 1:1000), GFAP (an anti‐GFAP, chicken polyclonal, Cat. PA1‐10004, Thermo Fisher Scientific, Massachusetts, USA, 1:1000), Iba1 (an anti‐Iba1, goat polyclonal, Cat. 5076, Abcam, Cambridge, UK, 1:1000), PTN (an anti‐PTN, mouse monoclonal, H‐6 clone, Cat. sc‐74 443, Santa Cruz Biotechnology, Dallas, Texas, USA, 1:200), MK (an anti‐MK, mouse monoclonal, A‐9 clone, Cat. sc‐46 701, Santa Cruz Biotechnology, Dallas, Texas, USA, 1:200), and RPTPβ/ζ (an anti‐RPTP, rabbit polyclonal, Cat. PA5‐101832, Thermo Fisher Scientific, Massachusetts, USA, 1:500) was performed overnight. After rinsing, sections were incubated with appropriate Alexa Fluor conjugated secondary antibodies, all diluted 1:5000 in blocking solution, for 2 h at RT (goat Alexa Fluor 546, goat Alexa Fluor 555 and donkey Alexa Fluor 488, Thermo Fisher Scientific, Massachusetts, USA). Sections were mounted with DAPI‐containing fluoroshield mounting medium (ab104139, Abcam, Cambridge, UK). Images were acquired with the Automated Upright Microscope System (Leica, DM5500B). Quantification was performed following the protocol described in [35]. Briefly, three animals per group and 2–3 randomly selected fields in the lumbar SC per animal were analyzed. For astrogliosis and microglial activation, number of double‐stained GFAP^+^DAPI^+^ and Iba1^+^DAPI^+^ cells, respectively, were quantified per field, and the result was expressed as number of cells per mm^2^. For specific expression of PTN in neurons, the total number of triple‐stained PTN^+^NeuN^+^DAPI^+^ cells was divided by the total number of double‐stained NeuN^+^DAPI^+^ cells and data was expressed as the percentage of triple‐stained cells over double‐stained cells (PTN^+^NeuN^+^DAPI^+^/NeuN^+^DAPI^+^ cells × 100) per mm^2^. For specific expression of PTN in astrocytes, the total number of triple‐stained PTN^+^GFAP^+^DAPI^+^ cells was divided by the total number of double‐stained GFAP^+^DAPI^+^ cells, and data was expressed as the percentage of triple‐stained cells over double‐stained cells (PTN^+^GFAP^+^DAPI^+^/GFAP^+^DAPI^+^ cells × 100) per mm^2^. For specific expression of PTN in microglia, the total number of triple‐stained PTN^+^Iba1^+^DAPI^+^ cells was divided by the total number of double‐stained Iba^+^DAPI^+^ cells and data was expressed as the percentage of triple‐stained cells over double‐stained cells (PTN^+^Iba^+^DAPI^+^/Iba1^+^DAPI^+^ cells × 100) per mm^2^. The same procedure was conducted for the specific expression of MK and RPTPβ/ζ in neurons, astrocytes, and microglia. Number of cells were measured using ImageJ Cell Counter plugin.

### 2.6 Nissl Staining

Lumbar SC sections were attemperated and then immersed in a 1:1 ethanol/chloroform solution for 30 min. Subsequently, the sections were subjected to 100%, 95%, 70%, and 50% ethanol for 2 min each, and washed in distilled water for 5 min. The sections were then stained with 0.1% cresyl violet solution for 6 min. Following staining, sections were rinsed in distilled water and subjected to differentiation at 50%, 95%, and 100% ethanol, followed by incubation in xylene for 3 min. Finally, the sections were mounted with DPX. Pictures of each section from L1 to L6 were taken using the Automated Upright Microscope System (Leica, DM5500B). MNs were identified as dark‐violet‐stained cells with a maximum diameter greater than 20 μm, located in the ventral horn of the SC.

### 2.7 Statistical Analysis

Data are presented as means ± standard error of the mean (SEM). For gene and protein expression analysis, two‐way analysis of variance (ANOVA) was used to assess the effects of two independent factors. When appropriate, Tukey's post hoc test was used for multiple comparisons. For immunofluorescence signal analysis, two‐sample Student's *t*‐test was performed. *p* values less than 0.05 were considered significant.

## 3 Results

### 3.1 PTN, MK, and RPTP Gene and Protein Expression Is Upregulated at the End Stage of Disease in the Lumbar SC of TDP‐43^A315T^ Mice

**FIGURE 1:**
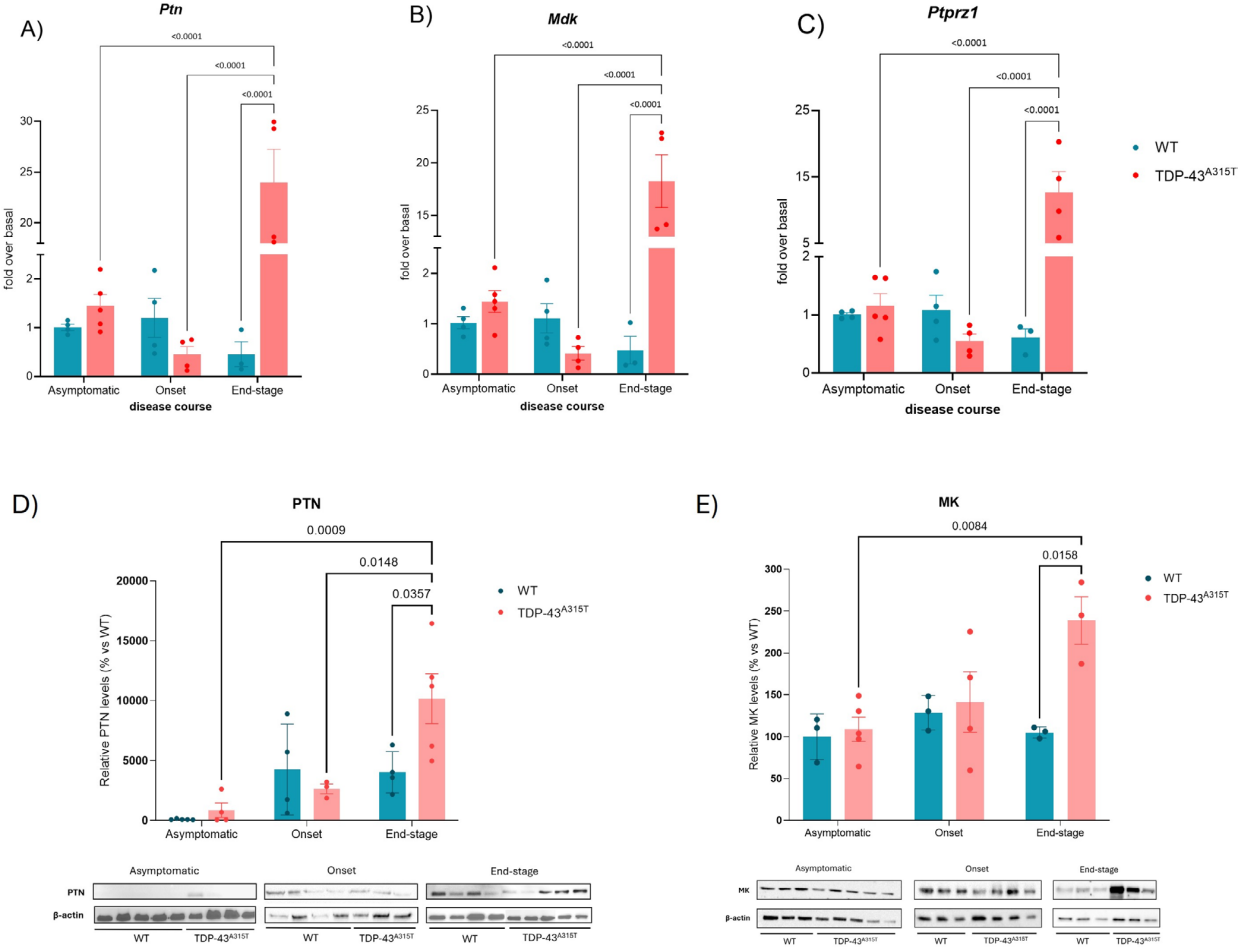
Gene and protein expression analysis in the lumbar spinal cord of TDP‐43^A315T^ and age‐matched WT mice at 42 (asymptomatic), 65 (onset), and 100 days (end‐stage) by quantitative real‐time PCR of *Ptn* (A), *Mdk* (B), and *Ptprz1* (C), and western blotting of PTN (D) and MK (E). Gene expression levels were normalized to 18S rRNA (*n* = 4–5 mice per group). Protein expression was normalized to β‐actin (*n* = 3–5 mice per group). Data is presented as mean ± SEM. Statistical analysis was performed using two‐way ANOVA.

Given the limited understanding of how the PTN pathway is involved in the progression of ALS, we aimed to address this gap by performing a comprehensive molecular characterization of their gene and protein expression patterns in the lumbar SC across the three stages of the disease in TDP‐43^A315T^ mice, compared to age‐matched WT littermates, using RT‐qPCR and WB analysis (Figure 1). In our study, asymptomatic samples from male TDP‐43^A315T^ mice were typically collected at 42–45 days of age, prior to the onset of symptoms, which usually appear around 65–70 days of age. Symptomatic animals were defined based on the presence of these clinical signs, and the end‐stage was reached approximately 2–4 weeks after symptom onset, depending on individual variability, but generally between 95 and 100 ± 2 days of age [36].

RT‐qPCR analysis revealed significant disease‐stage‐dependent changes in gene expression. Specifically, *Ptn*, *Mdk*, and *Ptprz1* mRNA levels were significantly upregulated (*p* < 0.001) at the end‐stage of disease compared to WT controls. At this stage, *Ptn* expression increased by approximately 24‐fold (Figure 1A), *Mdk* by 18‐fold (Figure 1B), and *Ptprz1* (Figure 1C) by 14‐fold relative to WT levels. Moreover, within the TDP‐43^A315T^ mice, a significant increase in the expression of all three genes was observed at the end‐stage compared to both the asymptomatic and onset stages (Figure 1), suggesting an upregulation that correlates with disease severity. WB analysis showed that both PTN and MK were overexpressed at the end‐stage of disease in TDP‐43^A315T^ mice, relative to time‐matched WT controls. PTN was significantly elevated at end‐stage compared to asymptomatic and onset stage in TDP‐43^A315T^ mice, while MK was found to be significantly increased at end‐stage compared to asymptomatic TDP‐43^A315T^ mice (Figure 1D,E). At the end‐stage of the disease, Nissl staining of the lumbar SC revealed that TDP‐43^A315T^ mice had ~20% fewer MNs compared with WT mice (Figure S1), confirming the presence of MN pathology characteristic of the terminal stage of the disease.

### 3.2 Expression Pattern of PTN, MK, and RPTPβ/ζ in the Lumbar SC of TDP‐43^A315T^ Mice at the End Stage of Disease

**FIGURE 2:**
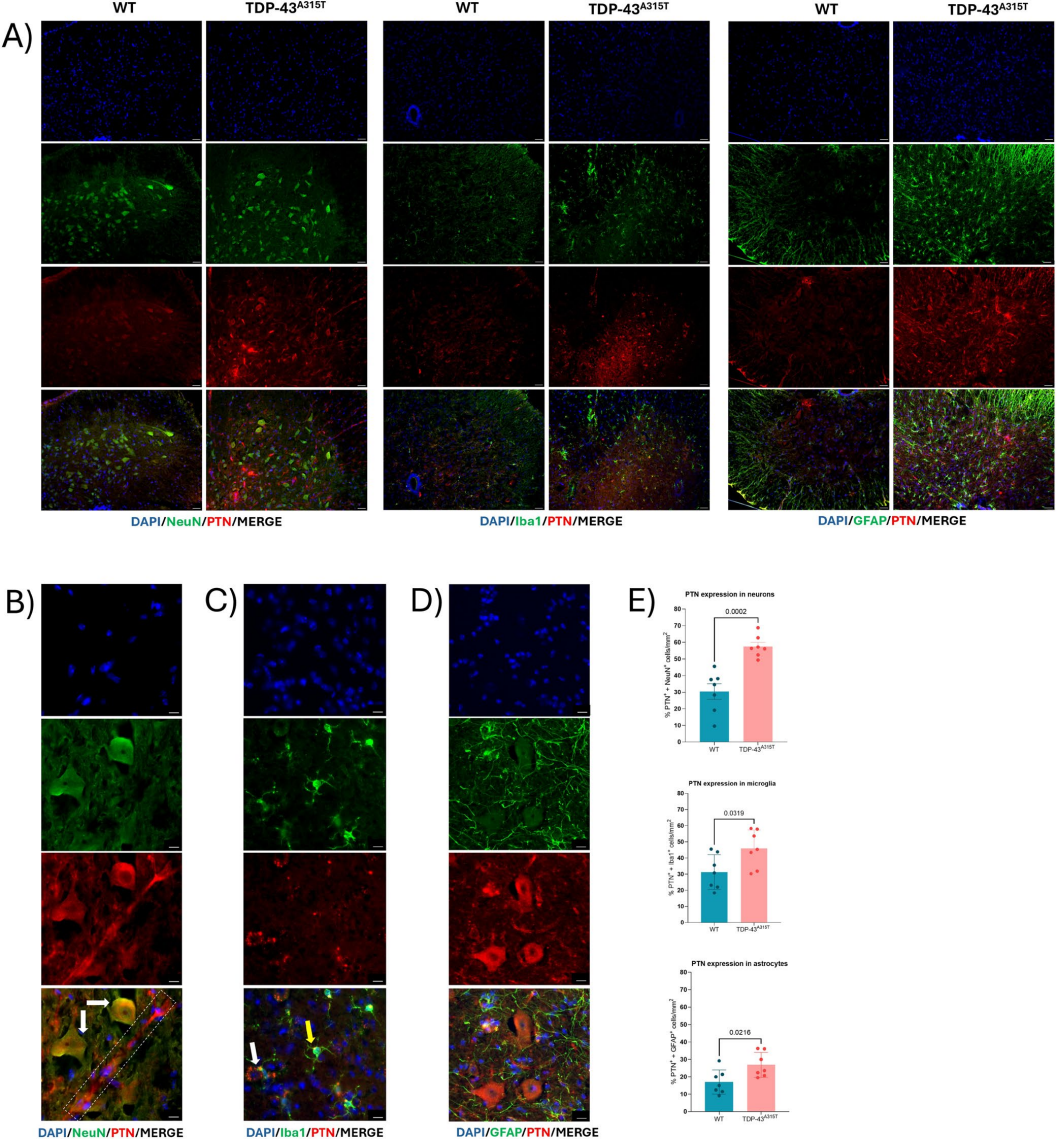
Immunofluorescence analysis of PTN expression in the lumbar SC of 100‐days‐old WT and age‐matched TDP‐43^A315T^ mice. Representative images of SC lumbar cross section stained with (A) DAPI (blue), NeuN, GFAP or Iba1 (green), and PTN (red) antibodies (×20 magnification). (B) DAPI (blue), NeuN (green), and PTN (red) antibodies (×100 magnification); white arrows indicate PTN expression in motor neurons; the dashed white rectangle highlights a microvasculature‐like structure exhibiting intense PTN immunoreactivity. (C) DAPI (blue), Iba1 (green), and PTN (red) antibodies (×100 magnification); white arrow indicates punctate PTN labeling within a motor neuron; yellow arrow indicates overlap of PTN and Iba1^+^ microglia. (D) DAPI (blue), GFAP (green), and PTN (red) antibodies (×100 magnification). Scale bars: 100 (A) and 50 μm (B–D). (E) Quantification of the number of neurons (NeuN^+^ cells), microglia (Iba1^+^ cells), and astrocytes (GFAP^+^ cells) with positive staining for PTN (*n* = 3 mice per group). Data is presented as mean ± SEM. Statistical analysis was performed using *t*‐test analysis.

**FIGURE 3:**
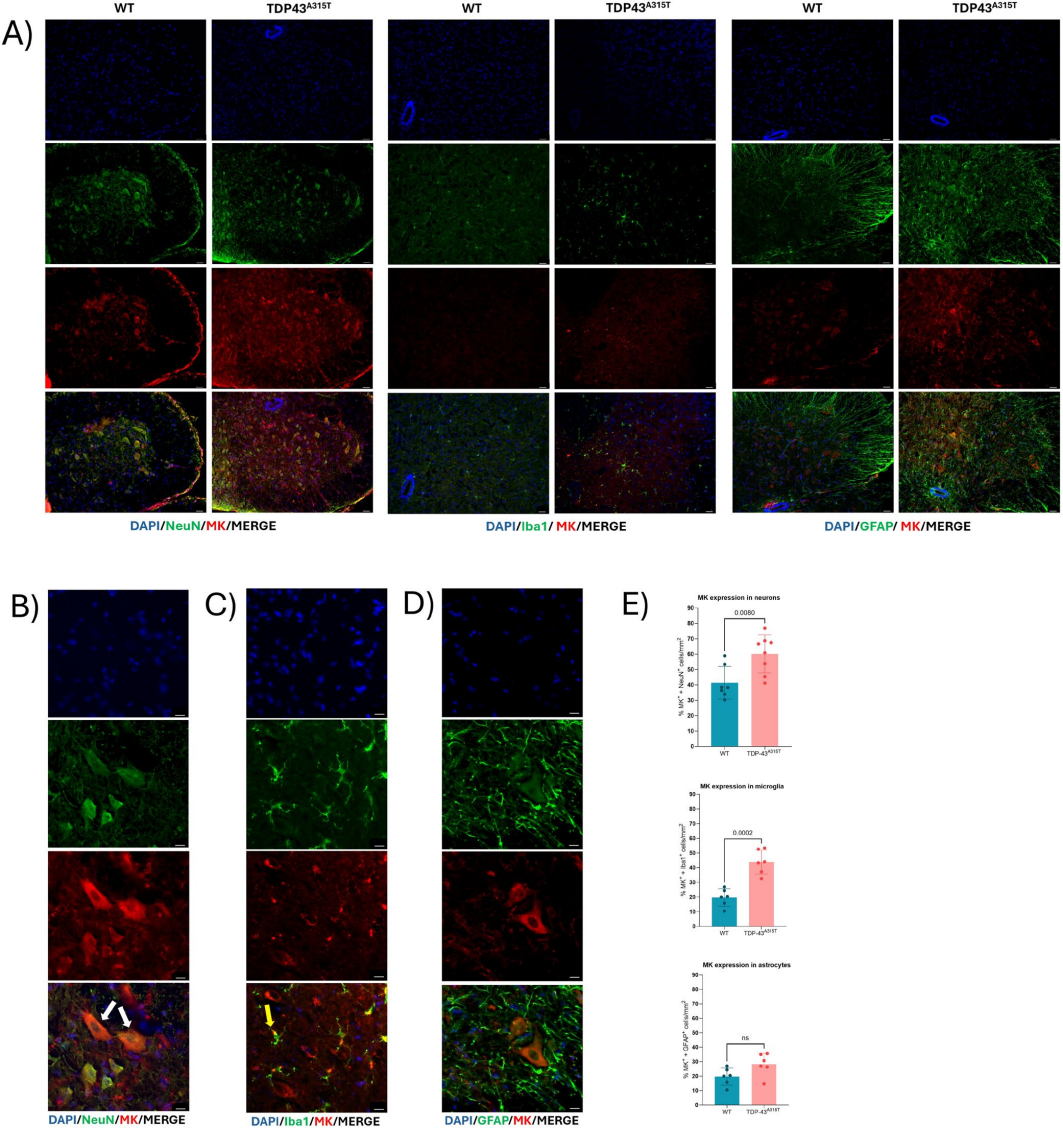
Immunofluorescence analysis of MK expression in the lumbar SC of 100‐days‐old WT and age‐matched TDP‐43^A315T^ mice. Representative images of SC lumbar cross section stained with (A) DAPI (blue), NeuN, GFAP or Iba1 (green), and MK (red) antibodies (×20 magnification). (B) DAPI (blue), NeuN (green), and MK (red) antibodies (×100 magnification); white arrows indicate expression of MK in motor neurons (white arrows). (C) DAPI (blue), Iba1 (green), and MK (red) antibodies (×100 magnification); yellow arrow indicates co‐localization of MK and Iba1 in microglia. (D) DAPI (blue), GFAP (green), and MK (red) antibodies (×100 magnification). Scale bars: 100 (A) and 50 μm (B–D). (E) Quantification of the number of neurons (NeuN^+^ cells), microglia (Iba1^+^ cells), and astrocytes (GFAP^+^ cells) with positive staining for MK (*n* = 3 mice per group). Data is presented as mean ± SEM. Statistical analysis was performed using *t*‐test analysis.

**FIGURE 4:**
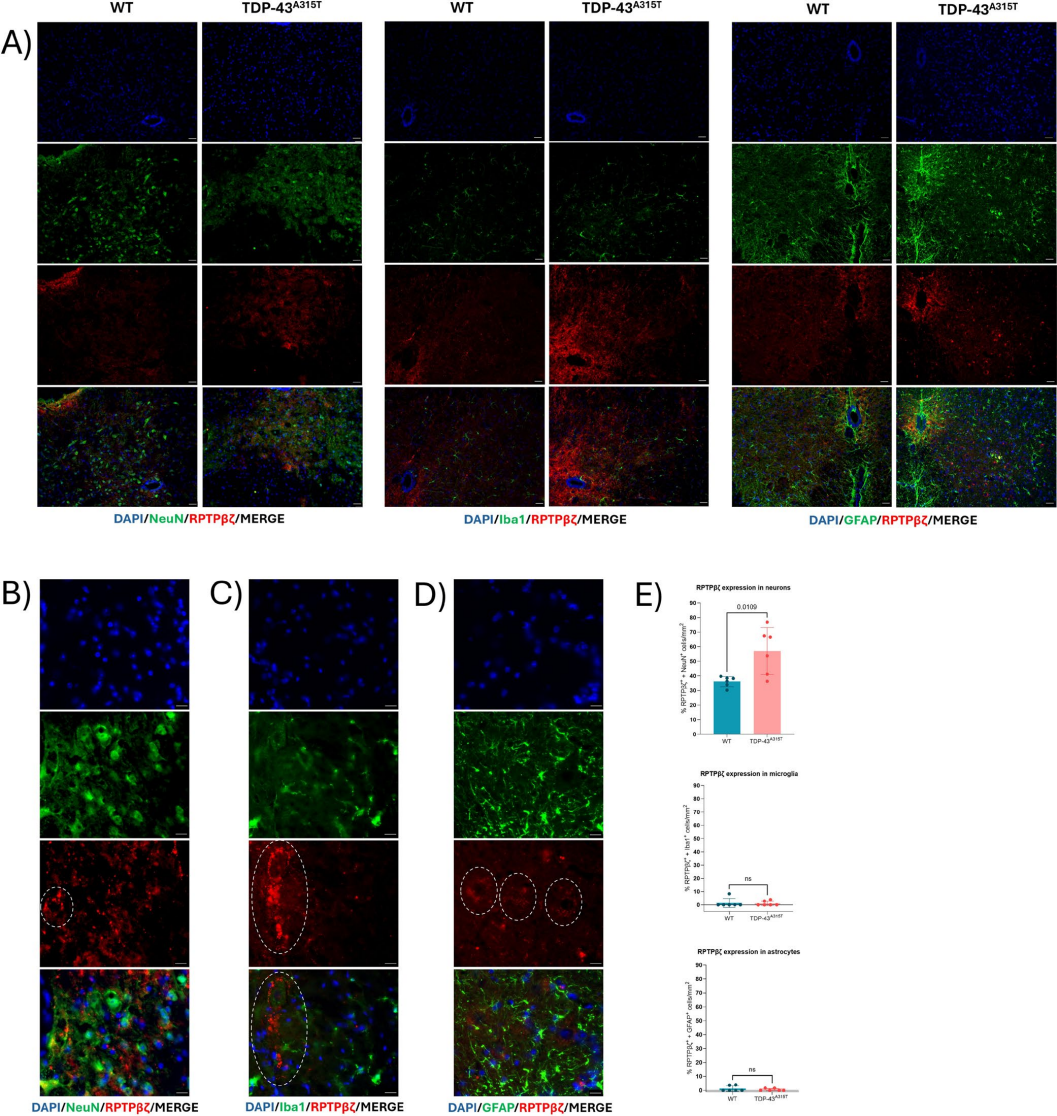
Immunofluorescence analysis of RPTPβ/ζ expression in the lumbar SC of 100‐days‐old WT and age‐matched TDP‐43^A315T^ mice. Representative images of SC lumbar cross section stained with (A) DAPI (blue), NeuN, GFAP or Iba1 (green), and RPTPβ/ζ (red) antibodies (×20 magnification). (B) DAPI (blue), NeuN (green), and RPTPβ/ζ (red) antibodies (×100 magnification); (C) DAPI (blue), Iba1 (green), and RPTPβ/ζ (red) antibodies (×100 magnification); (D) DAPI (blue), GFAP (green), and RPTPβ/ζ (red) antibodies (×100 magnification). Dashed white circles indicate expression of RPTPβ/ζ in motor neurons. Scale bars: 100 (A) and 50 μm (B–D). (E) Quantification of the number of neurons (NeuN^+^ cells), microglia (Iba1^+^ cells), and astrocytes (GFAP^+^ cells) with positive staining for RPTPβ/ζ (*n* = 3 mice per group). Data is presented as mean ± SEM. Statistical analysis was performed using *t*‐test analysis.

We next analyzed PTN, MK, and RPTPβ/ζ protein expression and localization at end‐stage of disease, corresponding to the time point at which both their mRNA and protein levels were found to be significantly upregulated. Immunofluorescence analysis of lumbar SC sections from TDP‐43^A315T^ mice revealed increased signs of neuroinflammation compared to WT controls, including increased activation of microglia and astrocytes, as indicated by elevated Iba1 and GFAP immunoreactivity, respectively (Figures 2A, 3A, and 4A and S2). PTN, MK, and RPTPβ/ζ proteins were detected in the lumbar SC of both WT and TDP‐43^A315T^ mice. However, PTN immunoreactivity was notably more intense in TDP‐43^A315T^ mice, exhibiting greatest expression within NeuN^+^ neurons, Iba1^+^ microglia, and GFAP^+^ astrocytes (Figure 2B–D), compared to WT mice (Figure S3). Strikingly, PTN also exhibited strong expression in perivascular structures resembling microvasculature‐associated pericytes, a pattern that was exclusively observed in TDP‐43^A315T^ mice (Figure 2B).

MK exhibited a similar expression pattern to PTN in the lumbar SC. In TDP‐43^A315T^ mice, MK was predominantly localized to NeuN^+^ neurons and Iba1^+^ microglia, with weaker expression detected in GFAP^+^ astrocytes (Figure 3A). MK immunolabeling was more prominent in TDP‐43^A315T^ than in WT mice, particularly within MN and microglial cells (Figures 3B,C and S4). Unlike PTN, MK did not display immunoreactivity in microvasculature‐like structures.

RPTPβ/ζ is mainly expressed in the dorsal horn of the SC, particularly within the white matter regions rich in myelinated axons, though moderate expression was also observed in the gray matter (Figure S5). RPTPβ/ζ immunoreactivity was markedly elevated in TDP‐43^A315T^ mice compared to WT animals (Figures 4A and S6) and was almost exclusively confined to NeuN^+^ neurons. Within these neurons, RPTPβ/ζ was detected both in the cytoplasm and in a punctate pattern consistent with membrane‐associated localization (Figure 4B–D).

## 4 Discussion

Biological bases of ALS are still largely unknown. Several pathogenic mechanisms have been identified as contributors to MN degeneration in ALS, including protein toxicity, oxidative damage, mitochondrial dysfunction, RNA metabolism disruption, impairment of DNA damage repair, and glutamate‐mediated excitotoxicity [1]. Increasing evidence indicates that neuroinflammation also contributes to MN injury and disease progression [37]. Consequently, strategies aimed at reducing neuroinflammation are emerging as promising therapeutic avenues. The identification of novel molecular targets involved in these processes is essential to advance the development of effective treatments for ALS.

PTN and MK are cytokines with established roles in both inflammation and regeneration, although their involvement in ALS has not previously been characterized. In this study, we investigated the expression of PTN, MDK, and their receptor RPTPβ/ζ in the lumbar SC of TDP‐43^A315T^ mice, a well‐characterized mouse model of ALS. We found significant upregulation of *Ptn*, *Mdk*, and *Ptprz1* gene expression at the end stage of the disease, accompanied by increased immunoreactivity of their encoded proteins. Analysis of PTN and MK protein expression also revealed similar upregulation at the end stage of the disease, suggesting that both cytokines may participate in ALS‐related inflammatory processes and/or in the degeneration‐regeneration dynamics of MNs. PTN is highly upregulated in acutely denervated nerves, which protects SC MN from chronic injury and promotes motor axon outgrowth [21]. Additionally, PTN promotes regeneration of myelinated axons in the peripheral nervous system [38]. Like PTN, MK increased its expression in damaged areas of traumatic SCI [39], and intrathecal administration of MK after SCI promotes functional recovery in rodent models [40].

Astrocyte and microglial activation is a hallmark of neuroinflammation and may have a dual role in neurodegenerative diseases [41]. Acute glial activation is essential for nervous system healing and regeneration, while sustained, chronic activation is implicated in the pathogenesis of neurodegenerative disorders. PTN amplifies microglial activation in response to acute stimuli and is upregulated in chronic neuroinflammatory conditions, where it modulates immune responses through its interaction with RPTPβ/ζ [26]. MK also participates in the recruitment and activation of immune cells during acute CNS inflammation, and in chronic neuroinflammatory processes MK mediates interactions between CNS‐infiltrating and resident cells, contributing to sustained inflammatory responses [42, 43]. In the present study, we show that the PTN/MK signaling axis is dysregulated in the lumbar SC of TDP‐43^A315T^ ALS mice. The marked overexpression of *Ptn*, *Mdk*, and *Ptprz1* mRNA and PTN and MK cytokines observed at the end stage of the disease aligns with the progressive neurodegeneration and neuroinflammatory responses in this model. TDP‐43 proteinopathies are characterized by the presence of hyperphosphorylated and ubiquitinated TDP‐43 inclusions in the cytoplasm. This results in neuronal toxicity, which ultimately leads to degeneration and inflammation of the neurons [44]. Thus, the upregulation of the PTN/MK pathway may represent either a maladaptive response or a compensatory neuroprotective mechanism, warranting further mechanistic investigation.

PTN and MK exert diverse effects through multiple receptors involved in cell proliferation, migration, inflammation, self‐renewal, and neuroplasticity, including syndecans, RPTPβ/ζ, ALK, Lrp1, nucleolin, neuropilin‐1, VEGFR2, and integrins [29, 45]. Among these, RPTPβ/ζ—mainly expressed in adult neurons and glia—is a key modulator of neuroinflammation and cell viability [46]. PTN and MK inhibit RPTPβ/ζ phosphatase activity, reducing phosphorylation of neuroinflammation‐related substrates, such as TrkA and Fyn kinase [47, 48]. Recently it has been shown that inhibition of RPTPβ/ζ decreases neuroinflammation in a mouse model of Alzheimer's disease [49]. The expression pattern of PTN, MK, and RPTPβ/ζ proteins was also investigated at the end‐stage of disease, when gene and protein expression was upregulated. Immunofluorescence analyses revealed increased PTN expression in neurons, microglia, and astrocytes at the end‐stage of the disease. Similarly to PTN, MK was shown to be increasingly expressed in neurons and microglia, while SC astrocytes exhibited a less pronounced MK signal. Notably, in our hands, RPTPβ/ζ displayed an almost exclusive neuronal expression pattern, where it localized both into the cytoplasm and in a membrane‐associated punctate pattern. These findings are consistent with a model in which PTN and MK may exert both autocrine effects in neurons and autocrine/paracrine actions in glial cells, suggesting a role in bidirectional neuron–glia communication [27]. Interestingly, PTN immunoreactivity was also detected in structures morphologically consistent with microvasculature‐associated pericytes, which are known to be a major source of PTN in the nervous system [35]. Although we did not employ specific pericyte markers, the observed PTN labeling in vascular‐like structures suggests potential involvement of pericytes in ALS pathology. Notably, pericyte loss has been reported in postmortem SC tissue from ALS patients, correlating with compromised barrier function [50]. The elevated PTN signal in these vascular‐like structures in TDP‐43^A315T^ mice may reflect an attempted neurovascular protective response.

In summary, our study uncovers, for the first time, a dysregulation of the PTN/MK signaling pathway in the SC of TDP‐43^A315T^ mice. The simultaneous upregulation of PTN, MK, and RPTPβ/ζ not only reveals a previously unrecognized molecular alteration in ALS but also suggests that this pathway may have a dual modulation of neuroinflammatory responses and neuroprotective mechanisms. These findings provide critical new insight into the molecular underpinnings of MN vulnerability and highlight the PTN/MK–RPTPβ/ζ axis as a compelling candidate for therapeutic targeting. Elucidating the precise regulatory mechanisms and functional consequences of this pathway may open new avenues for the development of interventions aimed at slowing disease progression or enhancing neuronal resilience. Future research should focus on defining the cell‐specific roles of PTN/MK signaling in ALS pathogenesis and exploring strategies to therapeutically modulate this pathway for maximal clinical benefit.

## 5 Study Design and Limitations

The objective of this study was to perform a primary characterization of PTN pathway in TDP‐43^A315T^ ALS. Samples used were obtained from those leftover samples from the study No. 26/OH 2018, where biochemical and pathological characterization of these mice were performed and published [36]. This approach is consistent with one of the 3Rs principles of animal research (Reduction) and ensured that tissues collected from sacrificed animals were used as efficiently as possible to obtain maximal experimental outcomes

## Funding

This work was supported by University San Pablo CEU‐Santander precompetitive grants (FUSPBS‐PPC03/2018 to M.J.P.M.) and Junta de Comunidades de Castilla‐la Mancha (SBPLY/17/180501/000303 to C.M.F.‐M.). Cristina Benito‐Casado is supported by a PhD Fellowship from the Consejería de Educación, Ciencia y Universidades Comunidad de Madrid (PIPF‐2023/SAL‐GL‐29613).

## Ethics Statement

Approval of the research protocol: Animal care and experimental procedures were conducted in accordance with the Spanish Guidelines for the Care and Use of Animals for Scientific Purposes and were approved by the Animal Ethics Committee of the Hospital Nacional de Parapléjicos in Toledo (Spain) (Approval No. 26/OH 2018).

## Consent

The authors have nothing to report.

## Conflicts of Interest

The authors declare no conflicts of interest.

## Supporting information


**Figure S1:** Nissl staining of the lumbar SC of 100‐days‐old WT and age‐matched TDP‐43^A315T^ mice. Representative images of a WT (A) and TDP‐43^A315T^ mice (B) cross section of lumbar SC, and quantification of motor neurons per section (C) (*n* = 2–3 mice per group). Data are presented as mean ± SEM. Statistical analysis was performed using two‐sample Student's *t*‐test.

**Figure S2:** Quantification of the number of astrocytes (GFAP^+^ + DAPI^+^ cells) (A) and microglia (Iba1^+^ + DAPI^+^ cells) (B) per mm^2^ in the lumbar SC of 100‐days‐old WT and age‐matched TDP‐43^A315T^ mice (*n* = 9 mice per group; 2–3 pictures per mice). Data are presented as mean ± SEM. Statistical analysis was performed using two‐sample Student's *t*‐test.

**Figure S3:** Immunofluorescence analysis of PTN expression in the lumbar SC of 100‐days‐old WT mice. Representative images of SC lumbar cross section stained with (A) DAPI (blue), NeuN (green), and PTN (red) antibodies; white arrows indicate PTN expression in motor neurons. (B) DAPI (blue), Iba1 (green) and PTN (red) antibodies; yellow arrow indicates overlap of PTN and Iba1^+^ microglia. (C) DAPI (blue), GFAP (green) and PTN (red) antibodies. Scale bars: 50 μm. ×100 magnification.

**Figure S4:** Immunofluorescence analysis of MK expression in the lumbar SC of 100‐days‐old WT mice. Representative images of lumbar SC cross section stained with (A) DAPI (blue), NeuN (green), and MK (red) antibodies; white arrows indicate MK expression in motor neurons. (B) DAPI (blue), Iba1 (green) and MK (red) antibodies; yellow arrow indicates overlap of MK and Iba1^+^ microglia. (C) DAPI (blue), GFAP (green), and MK (red) antibodies. Scale bars: 50 μm. ×100 magnification.

**Figure S5:** Immunofluorescence analysis of RPTPβ/ζ expression in the lumbar SC of 100‐days‐old WT and age‐matched TDP‐43^A315T^ mice. Representative images of lumbar SC cross section stained with DAPI (blue) and RPTPβ/ζ (red) antibodies. ×5 magnification.

**Figure S6:** Immunofluorescence analysis of RPTPβ/ζ expression in the lumbar SC of 100‐days‐old WT mice. Representative images of lumbar SC cross section stained with (A) DAPI (blue), NeuN (green), and RPTPβ/ζ (red) antibodies; white arrows indicate MK expression in motor neurons. (B) DAPI (blue), Iba1 (green), and RPTPβ/ζ (red) antibodies; yellow arrow indicates overlap of MK and Iba1^+^ microglia. (C) DAPI (blue), GFAP (green), and RPTPβ/ζ (red) antibodies. Scale bars: 50 μm. ×100 magnification.

## Data Availability

The datasets generated and/or analyzed during the current study will be made available in the institutional repository of Universidad San Pablo CEU upon publication of this article.
