# Supplementary figures and images for: Pleiotrophin/Midkine Pathway Is Dysregulated in a TDP‐43A315T Mouse Model of Amyotrophic Lateral Sclerosis (ALS)
Source: Neuropathology. 2026 Jan 28;46(1):e70044. doi: 10.1111/neup.70044 (PMC12851830; doi:10.1111/neup.70044)

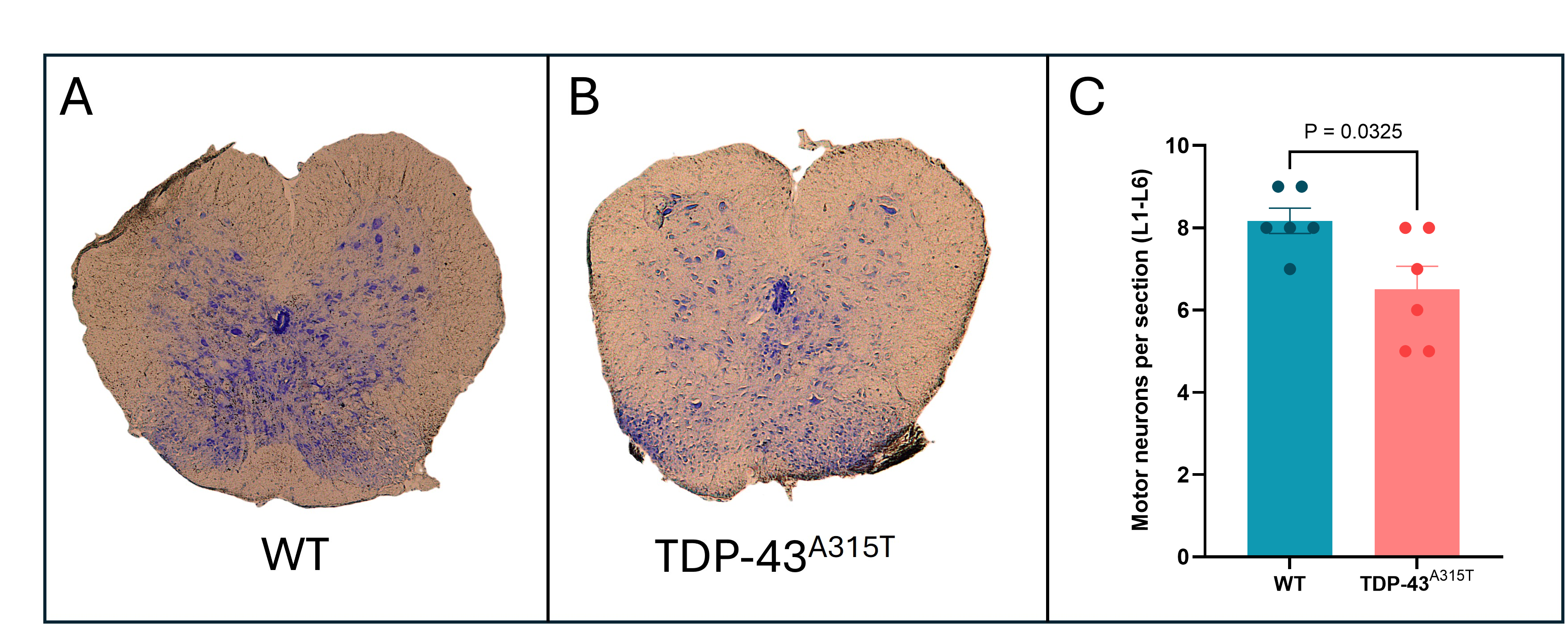

Supplement: Supplementary file 1 — Figure S1: Nissl staining of the lumbar SC of 100‐days‐old WT and age‐matched TDP‐43A315T mice. Representative images of a WT (A) and TDP‐43A315T mice (B) cross section of lumbar SC, and quantification of motor neurons per section (C) (n = 2–3 mice per group). Data are presented as mean ± SEM. Statistical analysis was performed using two‐sample Student's t‐test. [file NEUP-46-0-s004.png]

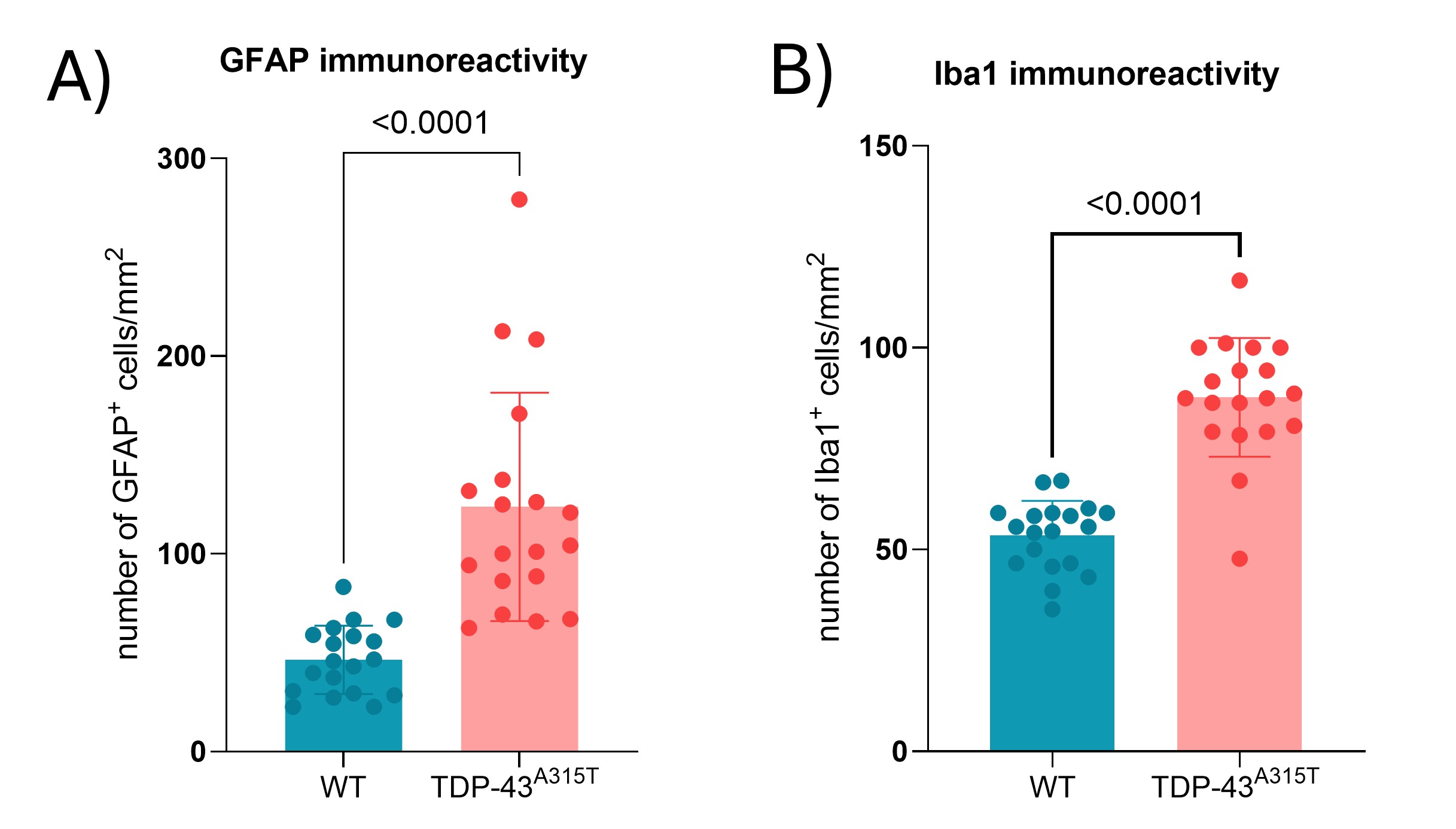

Supplement: Supplementary file 2 — Figure S2: Quantification of the number of astrocytes (GFAP+ + DAPI+ cells) (A) and microglia (Iba1+ + DAPI+ cells) (B) per mm2 in the lumbar SC of 100‐days‐old WT and age‐matched TDP‐43A315T mice (n = 9 mice per group; 2–3 pictures per mice). Data are presented as mean ± SEM. Statistical analysis was performed using two‐sample Student's t‐test. [file NEUP-46-0-s005.jpg]

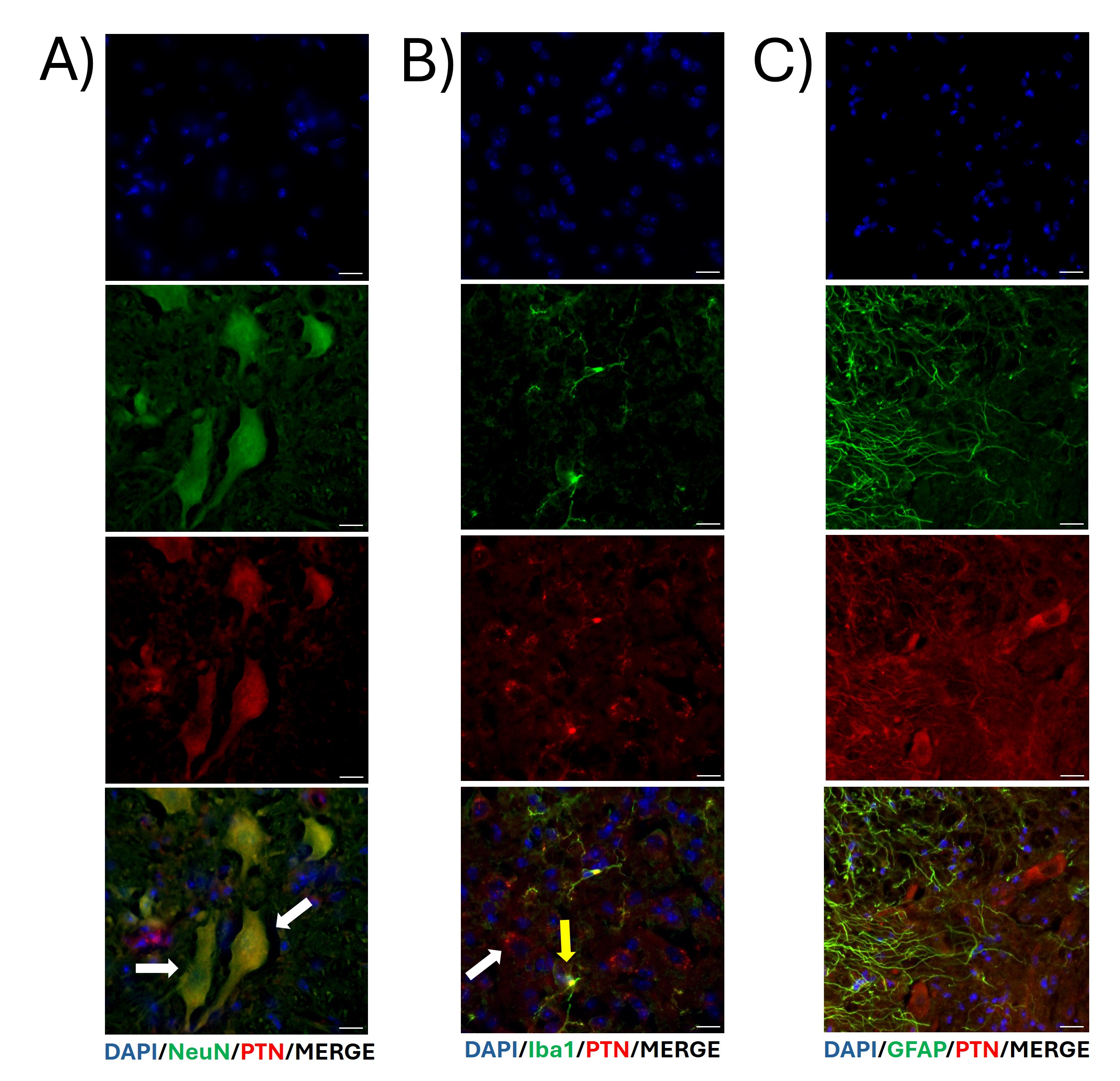

Supplement: Supplementary file 3 — Figure S3: Immunofluorescence analysis of PTN expression in the lumbar SC of 100‐days‐old WT mice. Representative images of SC lumbar cross section stained with (A) DAPI (blue), NeuN (green), and PTN (red) antibodies; white arrows indicate PTN expression in motor neurons. (B) DAPI (blue), Iba1 (green) and PTN (red) antibodies; yellow arrow indicates overlap of PTN and Iba1+ microglia. (C) DAPI (blue), GFAP (green) and PTN (red) antibodies. Scale bars: 50 μm. ×100 magnification. [file NEUP-46-0-s001.jpg]

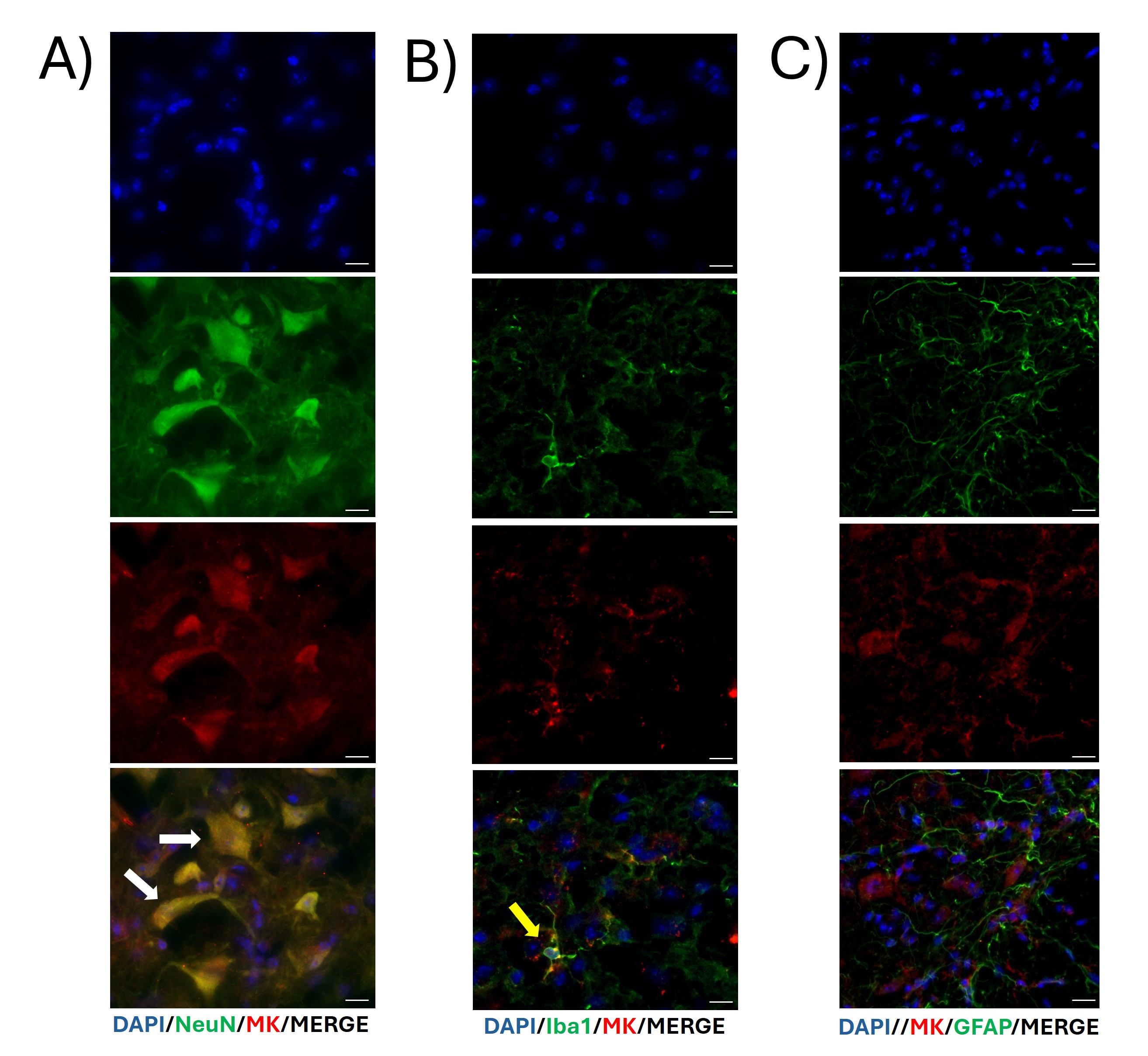

Supplement: Supplementary file 4 — Figure S4: Immunofluorescence analysis of MK expression in the lumbar SC of 100‐days‐old WT mice. Representative images of lumbar SC cross section stained with (A) DAPI (blue), NeuN (green), and MK (red) antibodies; white arrows indicate MK expression in motor neurons. (B) DAPI (blue), Iba1 (green) and MK (red) antibodies; yellow arrow indicates overlap of MK and Iba1+ microglia. (C) DAPI (blue), GFAP (green), and MK (red) antibodies. Scale bars: 50 μm. ×100 magnification. [file NEUP-46-0-s006.jpg]

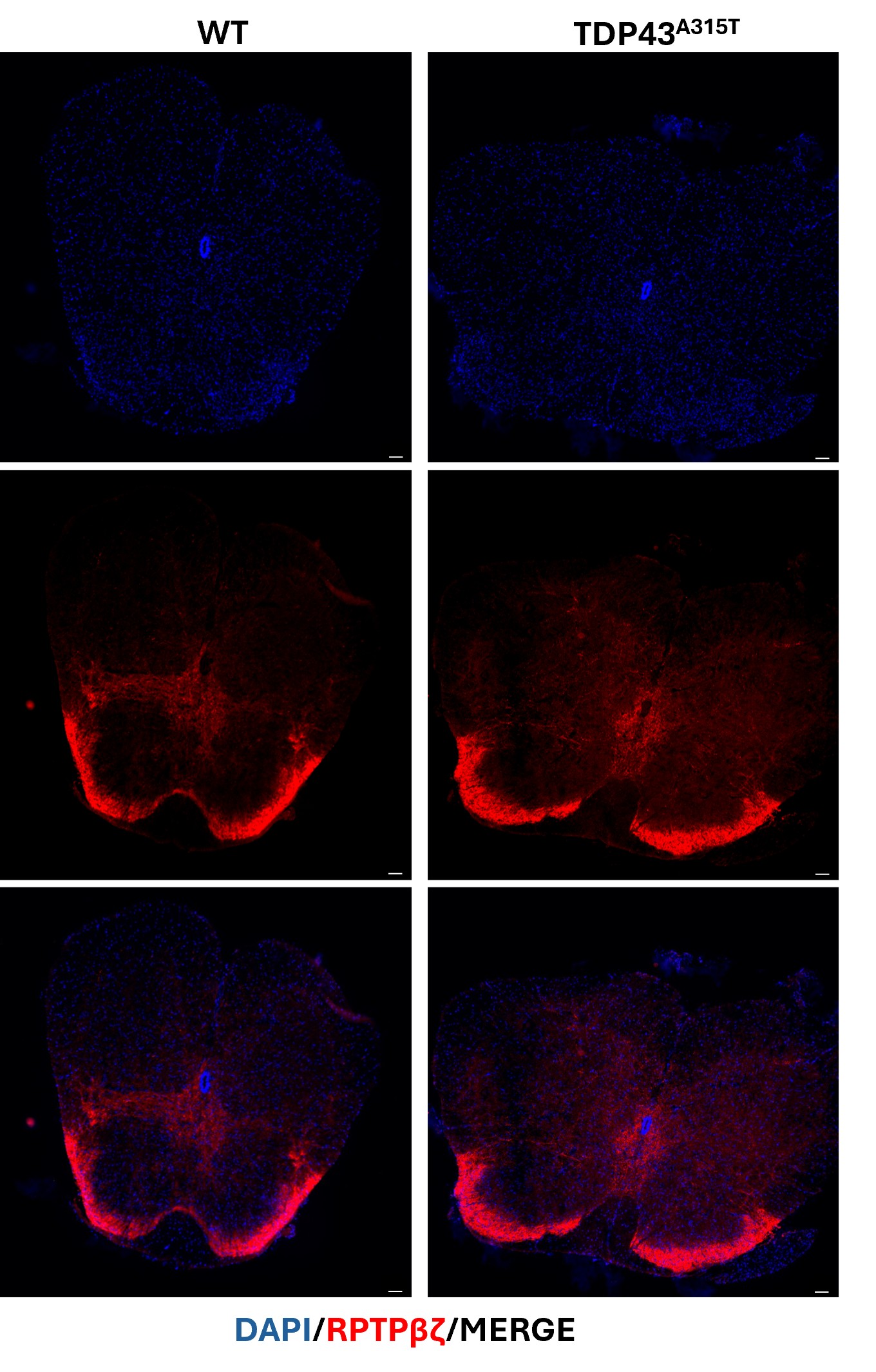

Supplement: Supplementary file 5 — Figure S5: Immunofluorescence analysis of RPTPβ/ζ expression in the lumbar SC of 100‐days‐old WT and age‐matched TDP‐43A315T mice. Representative images of lumbar SC cross section stained with DAPI (blue) and RPTPβ/ζ (red) antibodies. ×5 magnification. [file NEUP-46-0-s002.jpg]

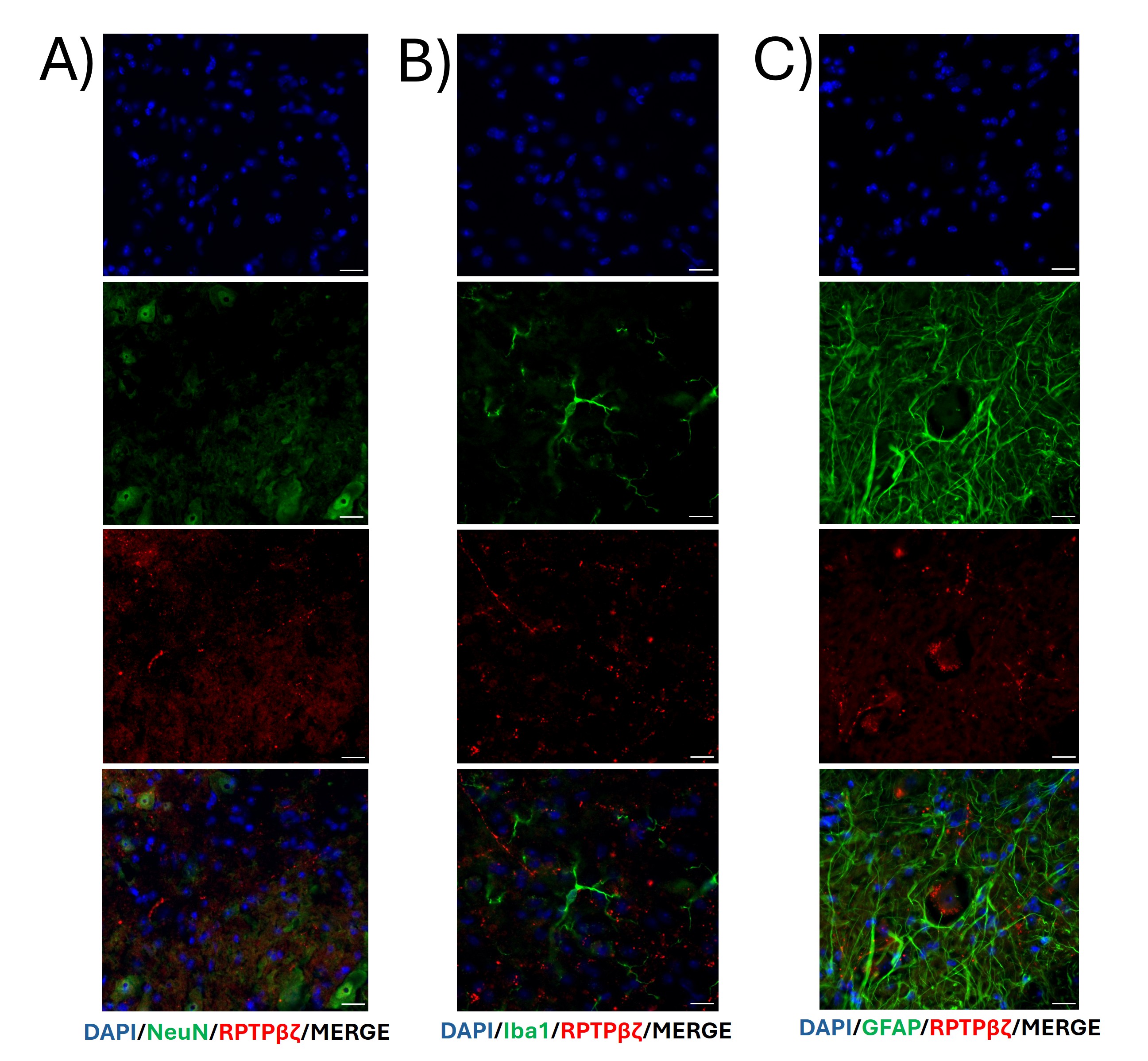

Supplement: Supplementary file 6 — Figure S6: Immunofluorescence analysis of RPTPβ/ζ expression in the lumbar SC of 100‐days‐old WT mice. Representative images of lumbar SC cross section stained with (A) DAPI (blue), NeuN (green), and RPTPβ/ζ (red) antibodies; white arrows indicate MK expression in motor neurons. (B) DAPI (blue), Iba1 (green), and RPTPβ/ζ (red) antibodies; yellow arrow indicates overlap of MK and Iba1+ microglia. (C) DAPI (blue), GFAP (green), and RPTPβ/ζ (red) antibodies. Scale bars: 50 μm. ×100 magnification. [file NEUP-46-0-s003.jpg]
